# Supplementary material for: Systematic design of cell membrane coating to improve tumor targeting of nanoparticles
Source: Nat Commun. 2022 Oct 19;13:6181. doi: 10.1038/s41467-022-33889-3 (PMC9580449; doi:10.1038/s41467-022-33889-3)
Supplement: Supplementary file 1 — Supplementary information [file 41467_2022_33889_MOESM1_ESM.pdf]

Supplementary Information for:

Systematic design of cell membrane coating to improve tumor targeting of nanoparticles

Lizhi Liu[1], Dingyi Pan[2], Sheng Chen[3], Maria-Viola Martikainen[4], Anna Kårlund[5], Jing Ke[6], Herkko Pulkkinen[1], Hanna Ruhanen[7,8], Marjut Roponen[4], Reijo Käkelä[7,8], Wujun Xu[1]*, Jie Wang[9]*, Vesa-Pekka Lehto[1]*

[1]Department of Applied Physics, University of Eastern Finland, 70210 Kuopio, Finland
[2]State Key Laboratory of Fluid Power and Mechatronic Systems, Department of Engineering Mechanics, Zhejiang University, Hangzhou 310027, China
[3]Department of Biomedical Engineering, Yale University, New Haven, CT, 06511, USA
[4]Department of Environmental and Biological Sciences, University of Eastern Finland, 70210 Kuopio, Finland
[5]Institute of Public Health and Clinical Nutrition, University of Eastern Finland, 70211 Kuopio, Finland
[6]Department of Chemistry, Boston College, Chestnut Hill, MA, 02467, USA
[7]Molecular and Integrative Biosciences Research Programme, Faculty of Biological and Environmental Sciences, University of Helsinki, 00014 Helsinki, Finland
[8]Helsinki University Lipidomics Unit (HiLIPID), Helsinki Institute of Life Science (HiLIFE) and Biocenter Finland, 00014 Helsinki, Finland
[9]School of Pharmacy, Anhui Medical University, Hefei 230032, China
*Corresponding author: wujun.xu@uef.fi; 2020500067@ahmu.edu.cn; vesa-pekka.lehto@uef.fi

Supplementary Figures

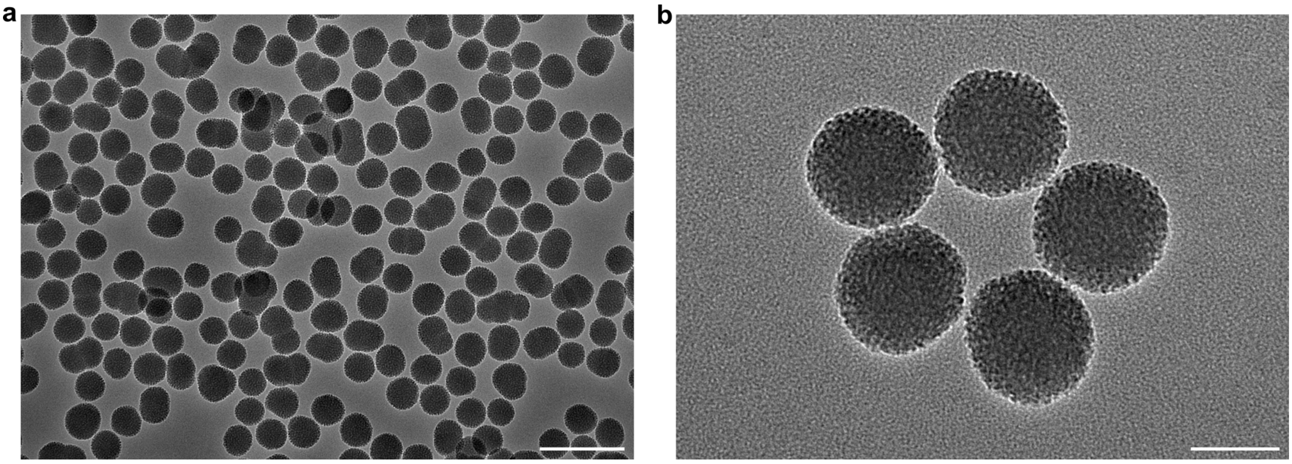

Supplementary Figure 1. TEM images of representative mesoporous SiO_2 NPs at low-magnification (**a**) and high-magnification (**b**). Scale bars, 200 nm in (a) and 50 nm in (b). Experiments were repeated three times independently with similar results.

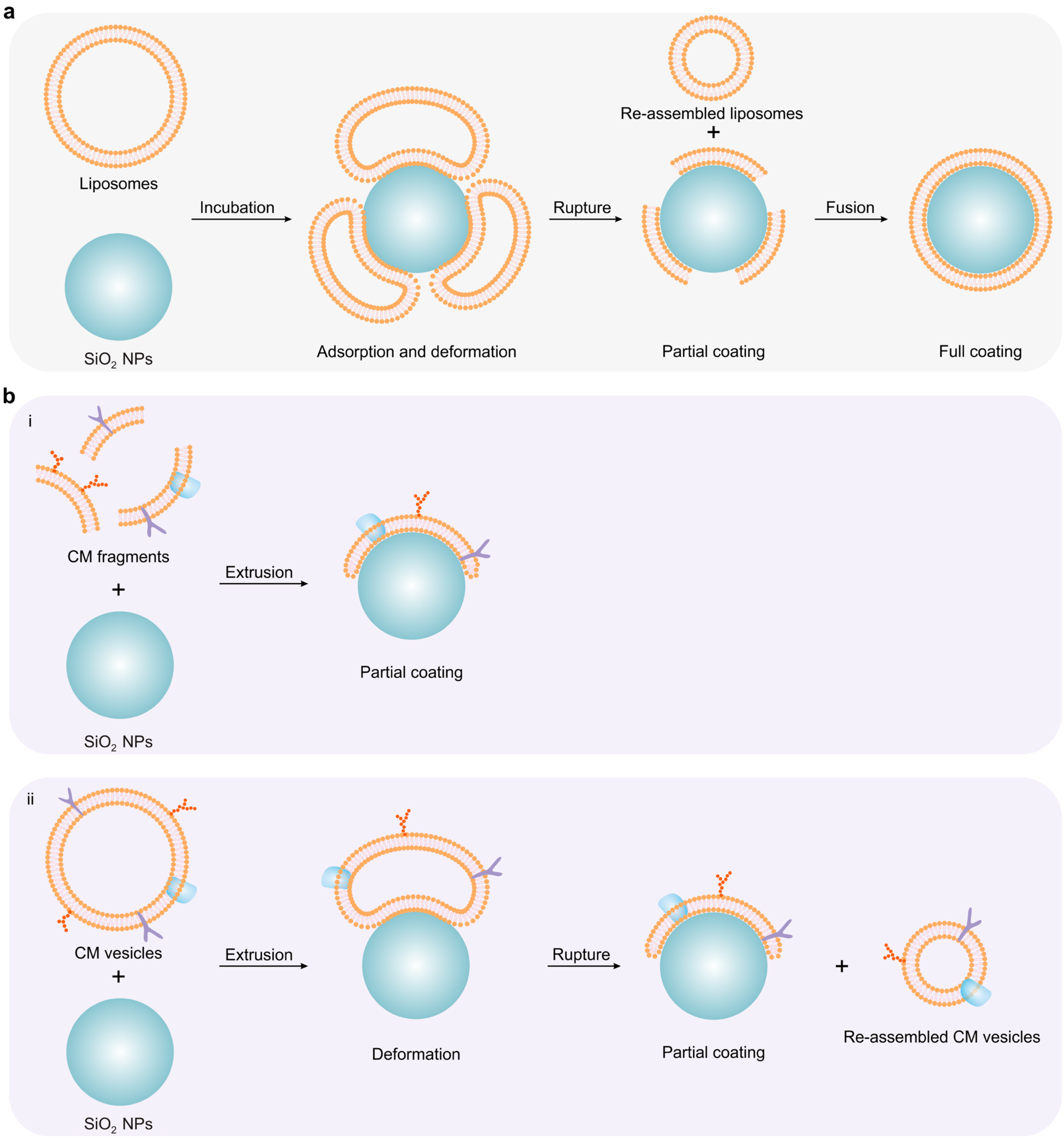

Supplementary Figure 2. a, Schematic drawing of the mechanism for the formation of LB-SiO_2 NPs. **b,** Partially coated CM-SiO_2 NPs could be resulted from the CM fragments adsorption (i) or the rupture of CM vesicles (ii) when co-extruded with SiO_2 NPs.

a b

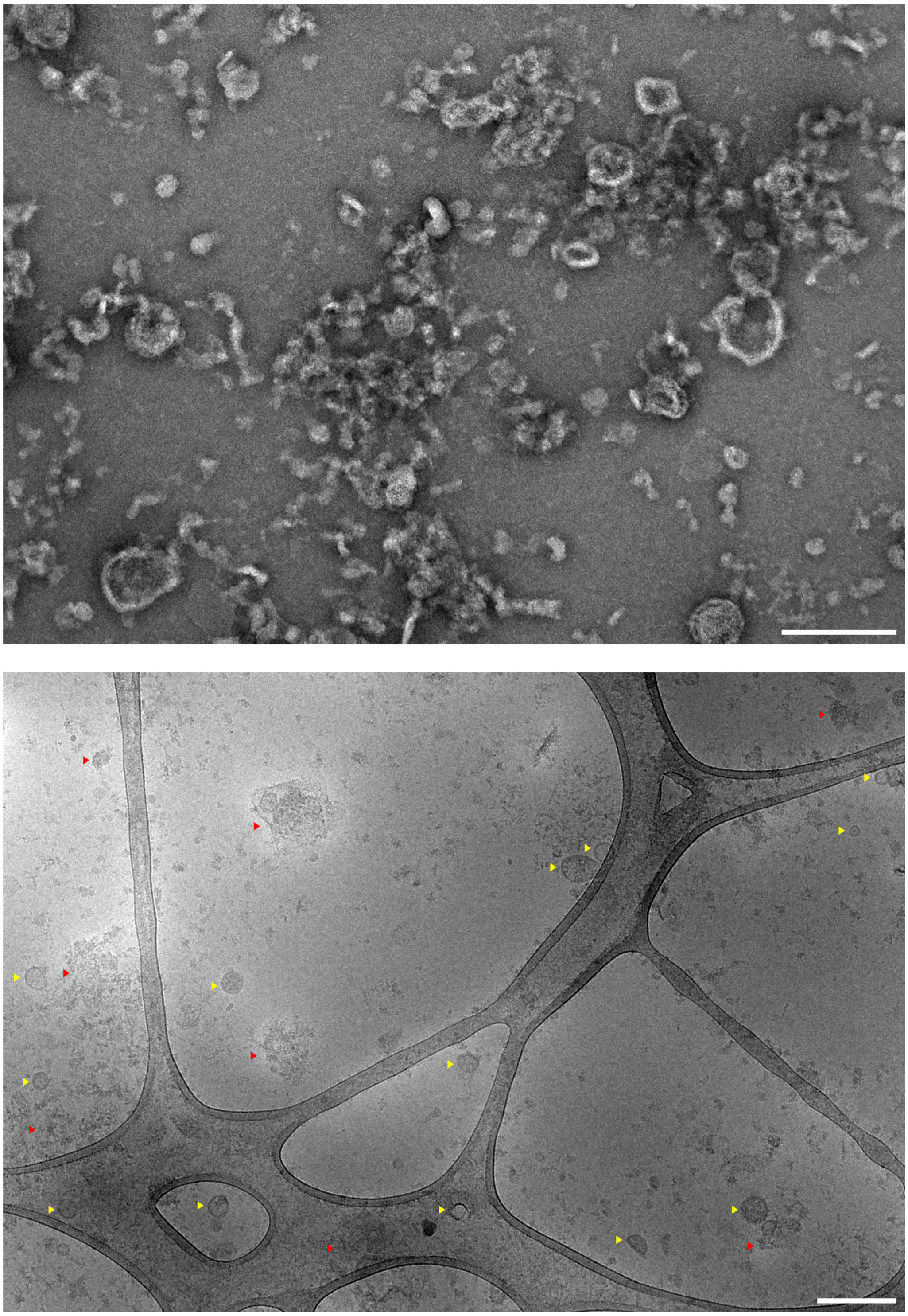

Supplementary Figure 3. TEM image (**a**) and Cryo-TEM image (**b**) of CM materials after extruded with a 200 nm PCTE membrane. The integrated CM vesicles and CM fragments in **b** are indicated by yellow and red arrowheads, respectively. Scale bars, 200 nm. Experiments were repeated three times independently with similar results.

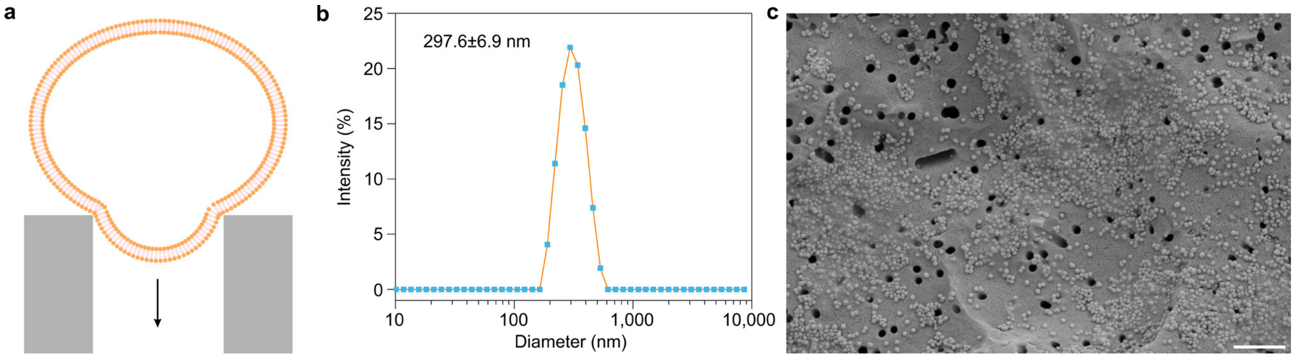

Supplementary Figure 4. a, Schematic illustration of a CM vesicle entering a pore of size with 200 nm that smaller than its diameter. **b,** Size distribution of CM vesicles with average diameter of 297.6 ± 6.9 nm, as measured by DLS. **c,** SEM image of PCTE membrane after co-extruded with CM vesicles (297.6 ± 6.9 nm) and SiO_2 NPs. Scale bar, 1 μm. Experiments in panels **b** and **c** were repeated three times independently with similar results. Source data are provided as a Source Data file.

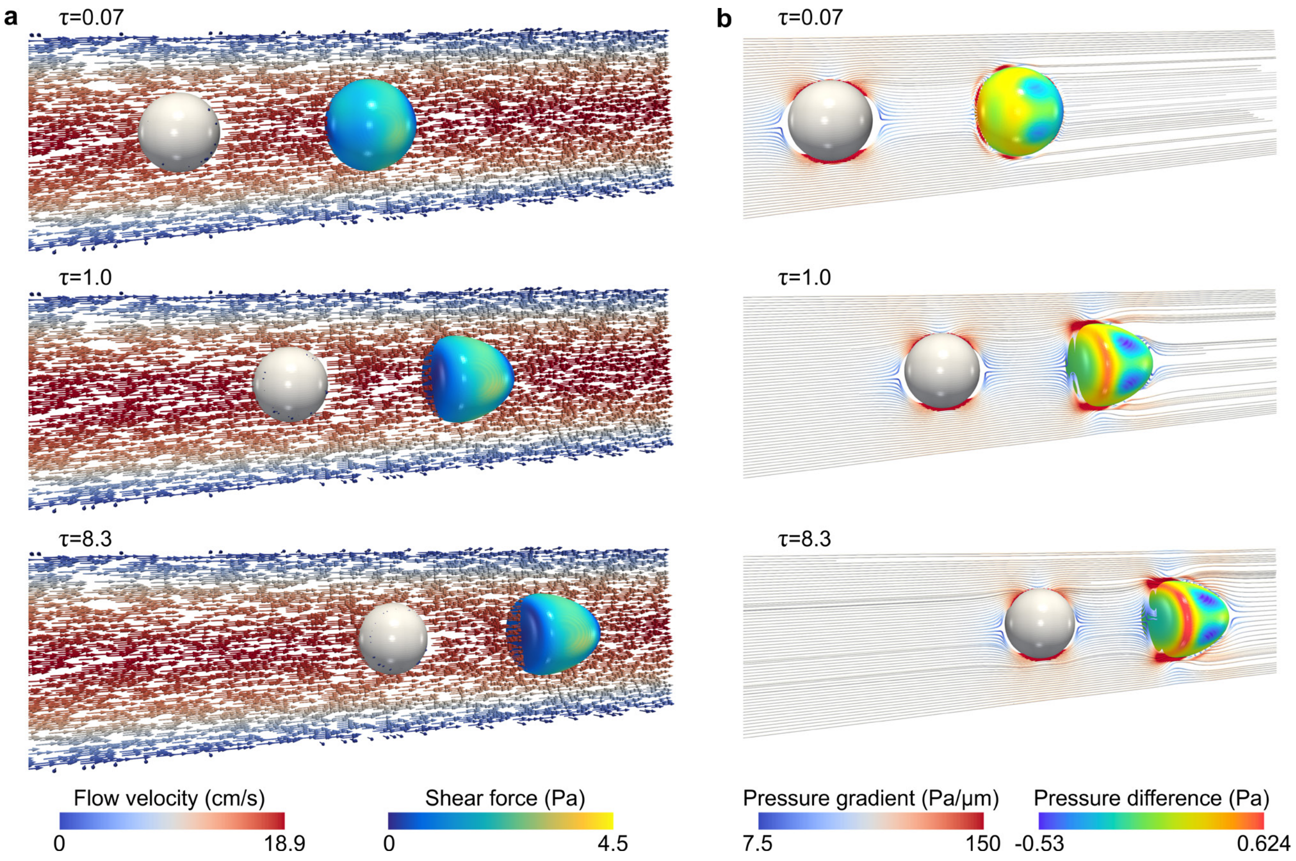

Supplementary Figure 5. Time evolution of CM vesicle deformation and positions with times taken as: τ = 0.07, τ = 1.0 and τ = 8.3. **a,** Distribution of the shear force along the surface of liposome with the background arrow field reflecting the surrounding fluid flow field. **b,** Distribution of the inner and outer pressure difference along the surface of liposome with the background field lines reflecting the pressure gradient of the flow.

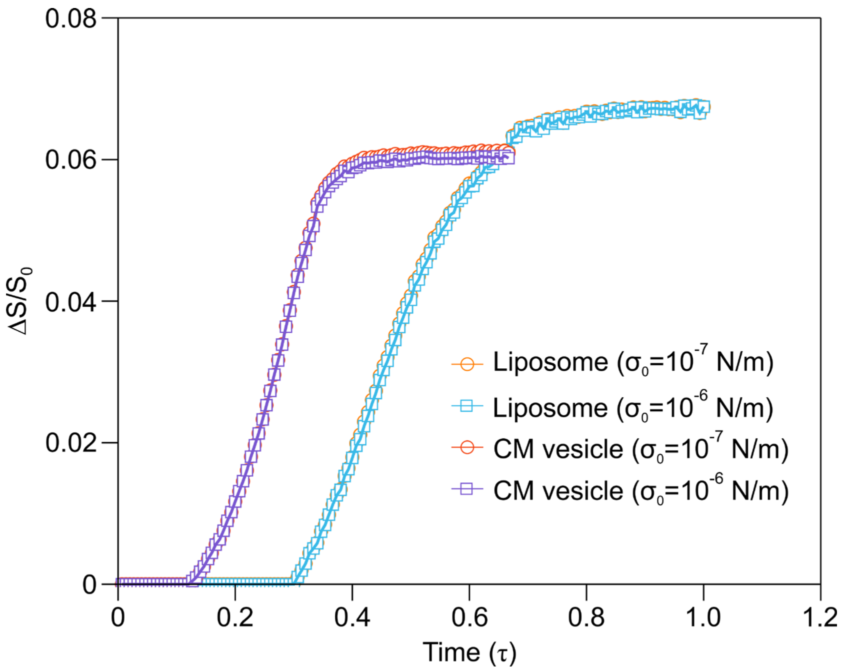

Supplementary Figure 6. Relative expansion of CM vesicle and liposome during extrusion. Two initial surface tensions (σ_0; 10^{-6} and 10^{-7} N/m) for CM vesicle and liposome were tested. Source data are provided as a Source Data file.

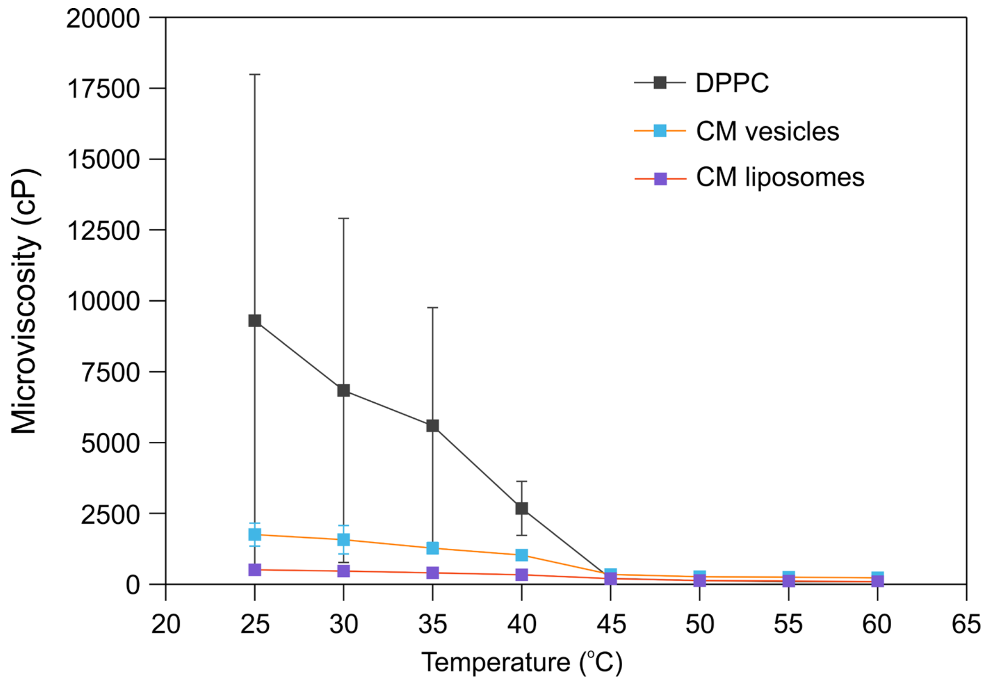

Supplementary Figure 7. The estimated microviscosity (η) values of DPPC liposomes, CM vesicles, and CM liposomes. Data represent the mean ± s.d. (*n* = 3 independent experiments). Source data are provided as a Source Data file.

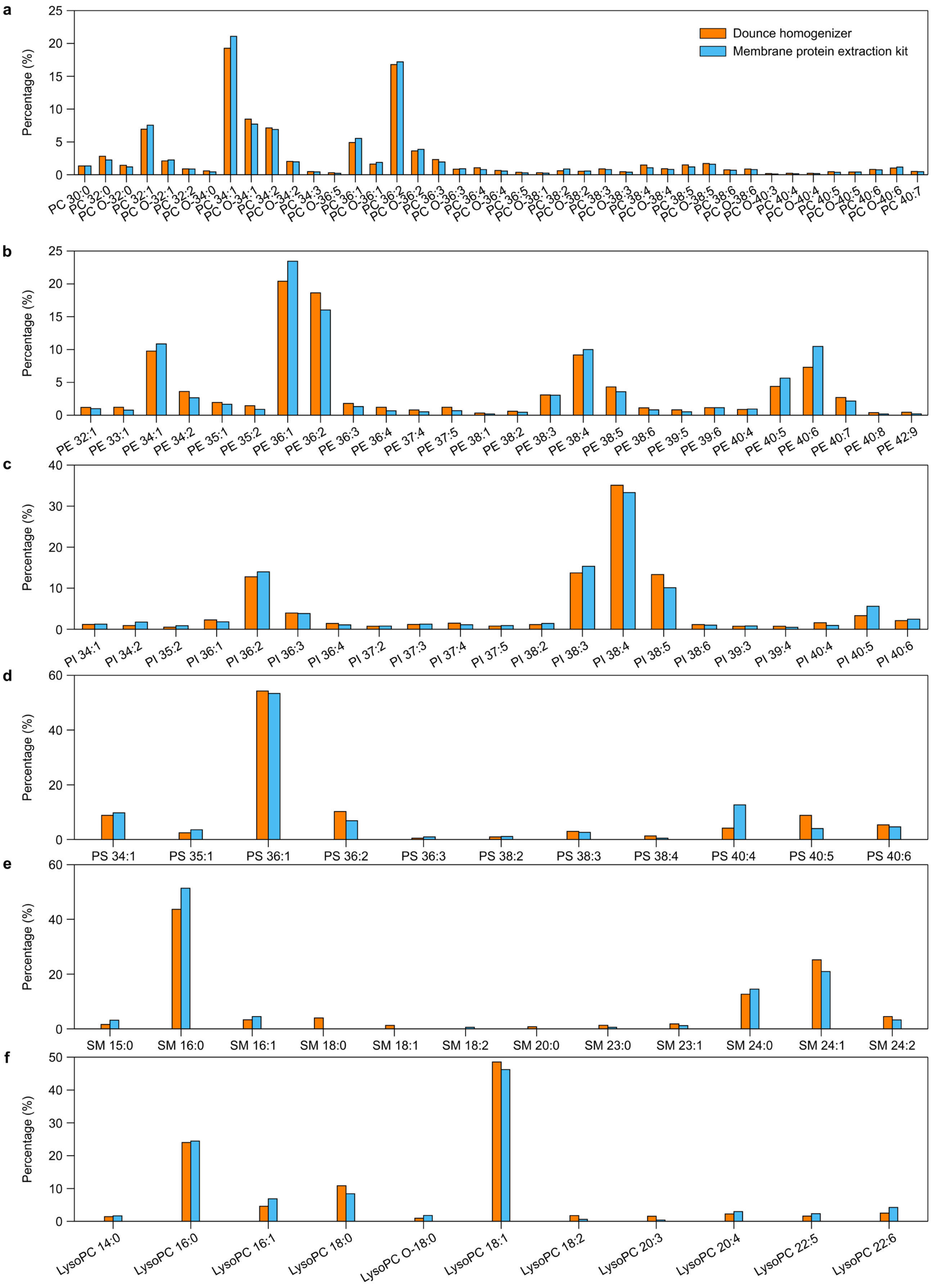

Supplementary Figure 8. Species profiles (mol%) of phosphatidylcholine (PC; **a**), phosphatidylethanolamine (PE; **b**), phosphatidylinositol (PI; **c**), phosphatidylserine (PS; **d**),

sphingomyelin (SM; **e**), and lysophosphatidylcholine (LysoPC; **f**) in the CM fragments obtained by the extraction method of Dounce homogenizer and membrane protein extraction kit. O- represents lipid species containing an ether chain. Experiments were repeated three times independently with similar results. Source data are provided as a Source Data file.

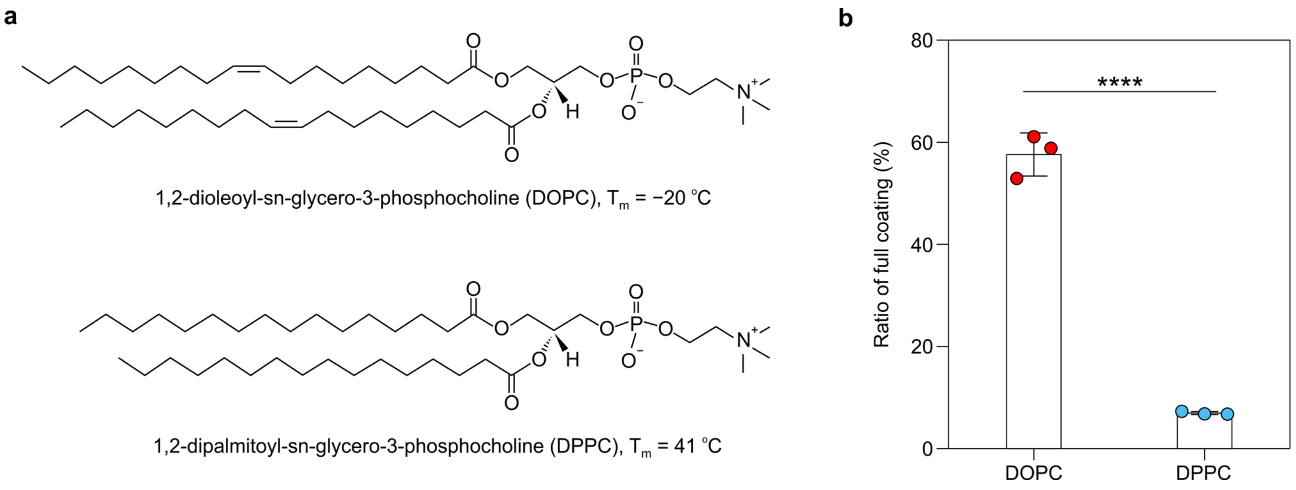

Supplementary Figure 9. a, Two liposomes composed of 1,2-dioleoyl-sn-glycero-3-phosphocholine (DOPC; T_m= −20 °C) and 1,2-dipalmitoyl-sn-glycero-3-phosphocholine (DPPC; T_m= 41 °C) were utilized to coat SiO_2 NPs. **b,** Quantification of the ratio of full LB coating with different liposomes (DOPC and DPPC), in which the coating process was performed at room temperature. Data represent the mean ± s.d. (n = 3 independent experiments). Significance was determined by one-way ANOVA followed by post hoc Tukey test. ****p < 0.0001. Source data are provided as a Source Data file.

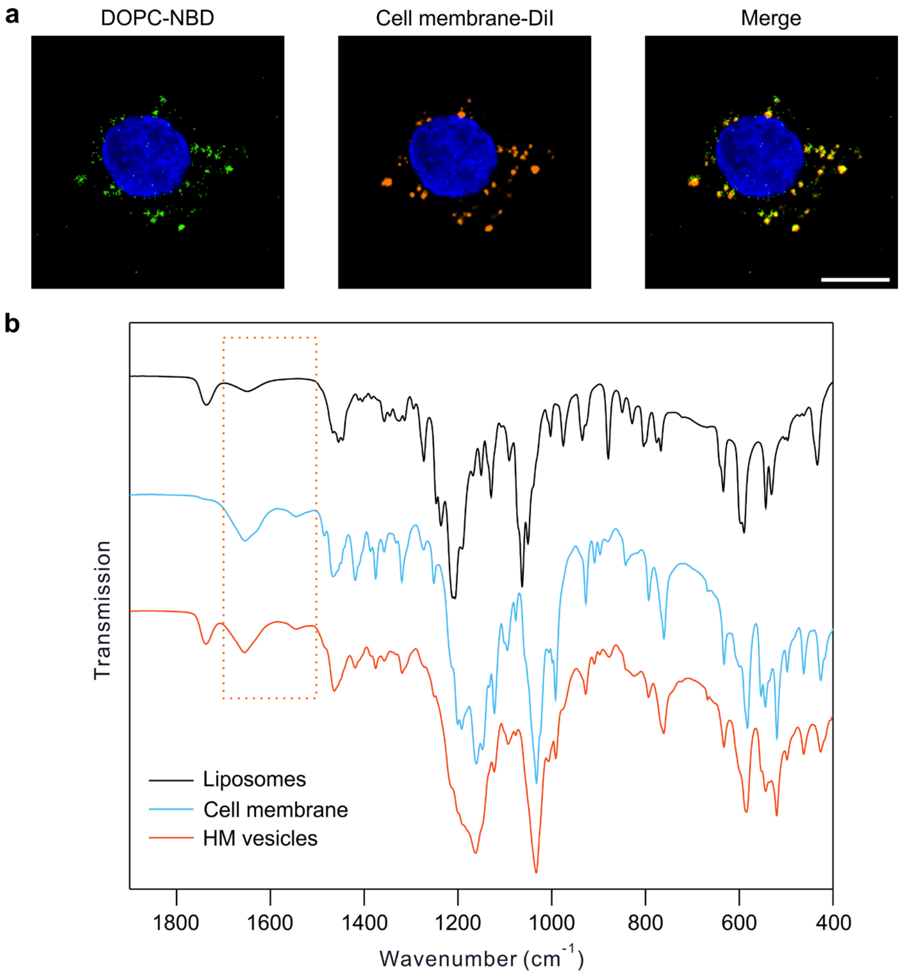

Supplementary Figure 10. a, Confocal laser scanning microscopy (CLSM) images showed the colocalization of the DOPC (labeled with NBD; green) and CM materials (labeled with DiI; orange) after being internalized by CT26 cells. The HM vesicles were incubated with CT26 cells for 4 h. The cell nuclei were stained with 4′,6-diamidino-2-phenylindole (DAPI) (blue). Scale bar, 10 μm. **b,** Representative Fourier transform infrared spectroscopy (FTIR, Thermo Nicolet iS 50) spectra of DOPC liposomes, CM, and HM vesicles showed a typical protein absorption band in the CM and HM vesicles, in which amide I band (1700-1600 cm^{-1}) was associated with C=O stretching vibrations, while amide II band (1600-1500 cm^{-1}) was due to the NH bending with C-N stretching vibrations. Experiments were repeated three times independently with similar results. Source data are provided as a Source Data file.

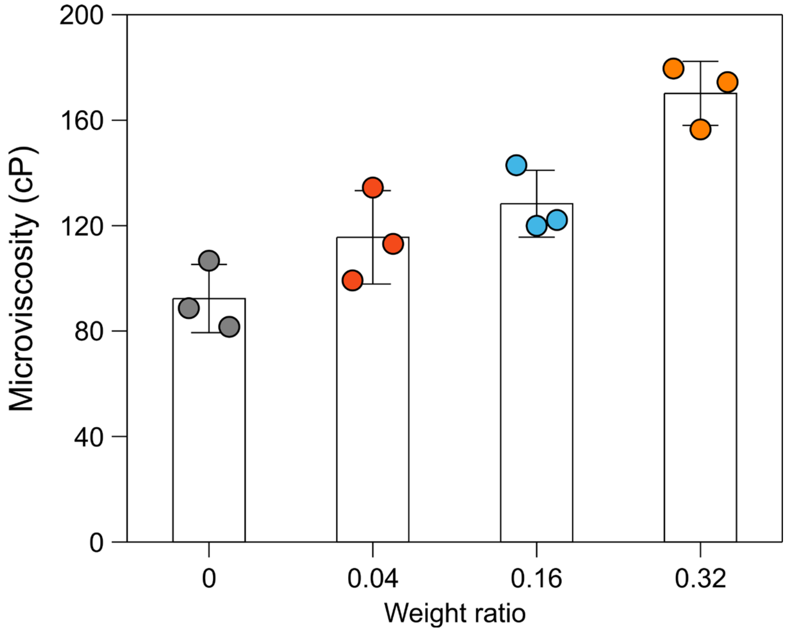

Supplementary Figure 11. The estimated microviscosity (η) values of HM vesicles prepared at different weight ratios (protein/lipid). Data represent the mean ± s.d. (n = 3 independent experiments). Source data are provided as a Source Data file.

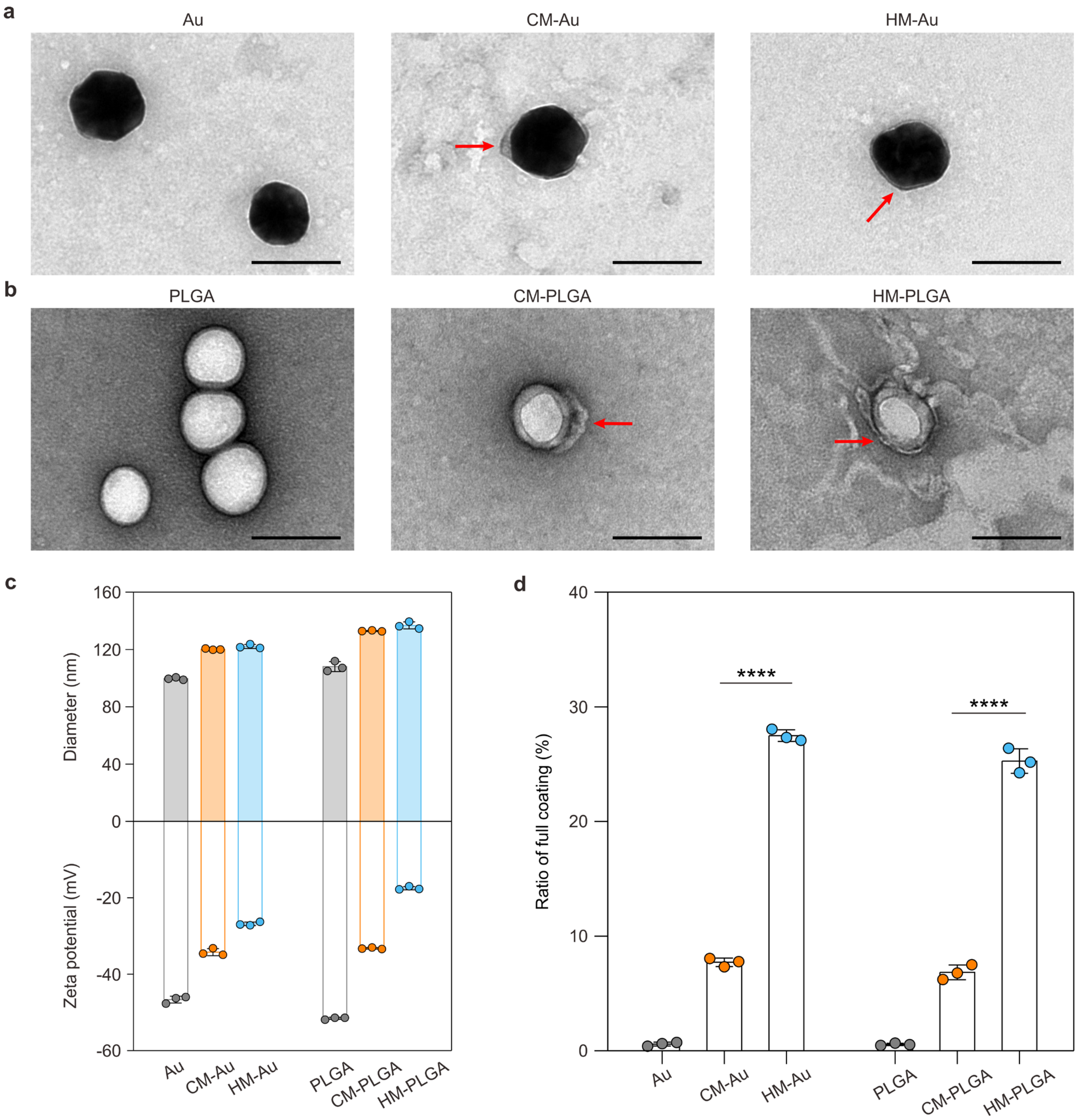

Supplementary Figure 12. a, TEM images of bare Au NPs, CM-Au NPs, and HM-Au NPs. Scale bars, 100 nm. **b,** TEM images of bare PLGA NPs, CM-PLGA NPs, and HM-PLGA NPs. Scale bars, 100 nm. The CM or HM coating layer is indicated as red arrows. **c,** Mean diameters and zeta potentials of Au NPs, CM-Au NPs, HM-Au NPs, PLGA NPs, CM-PLGA NPs, and HM-PLGA NPs. Data represent the mean ± s.d. ($n = 3$ independent experiments). **d,** Quantification of the ratio of full membrane coating for Au NPs, CM-Au NPs, HM-Au NPs, PLGA NPs, CM-PLGA NPs, and HM-PLGA NPs. Experiments in panels **a** and **b** were repeated three times independently with similar results. Data represent the mean ± s.d. ($n = 3$ independent experiments) in panels **c** and **d**. Significance was determined by one-way ANOVA followed by post hoc Tukey test. ****$p < 0.0001$. Source data are provided as a Source Data file.

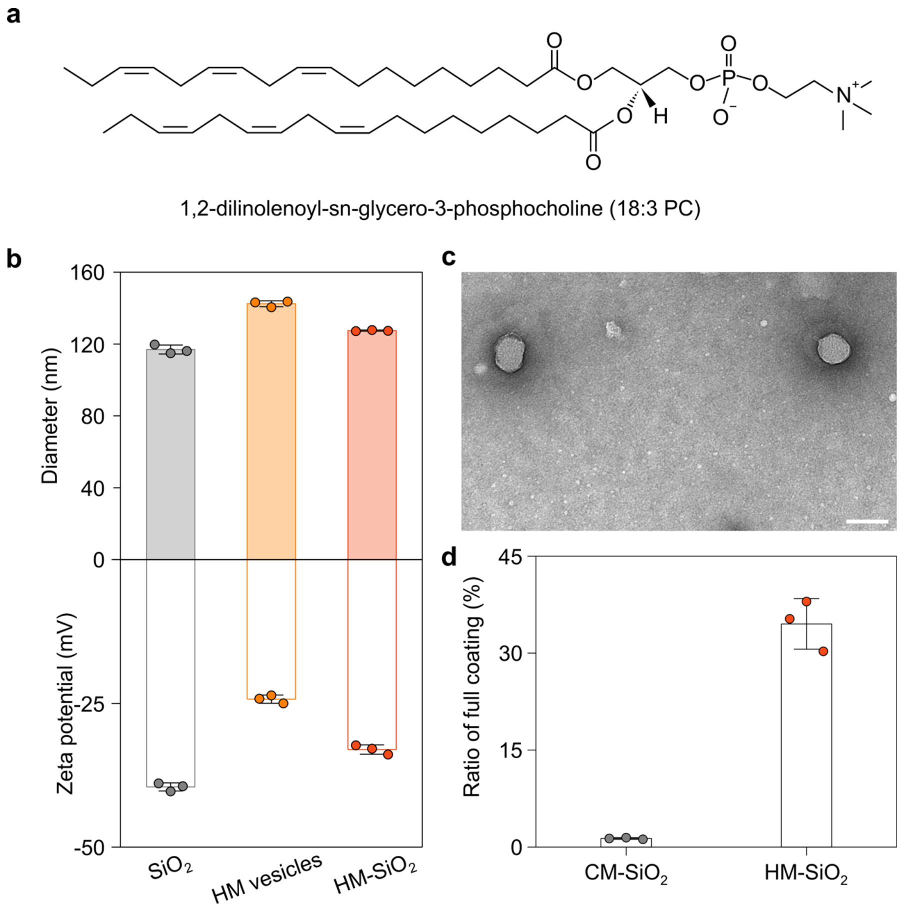

Supplementary Figure 13. a, Chemical structure of polyunsaturated 1,2-dilinolenoyl-sn-glycero-3-phosphocholine (18:3 PC) used in the HM coating. **b,** Mean diameters and zeta potentials of SiO_2 NPs, HM vesicles and HM-SiO_2 NPs, in which 18:3 PC was used as a helper phospholipid to prepare HM vesicles and HM coating. **c,** TEM image of HM-SiO_2 NPs. Scale bar, 100 nm. **d,** Quantification of the ratio of full CM coating for SiO_2 NPs coated with HM vesicles and the plain CM coating with the same amount of CM protein (0.4 mg/mL) used in the HM coating. Experiments in panel **c** were repeated three times independently with similar results. Data represent the mean ± s.d. (n = 3 independent experiments) in panels **b** and **d**. Source data are provided as a Source Data file.

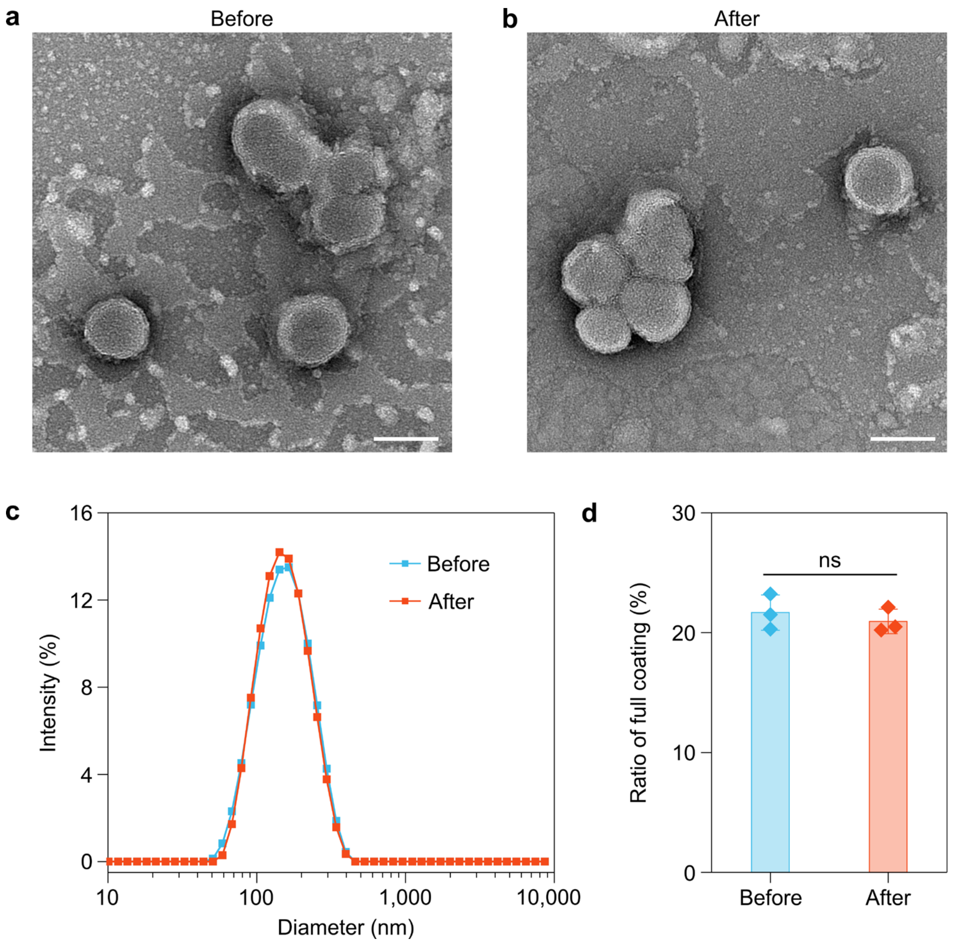

Supplementary Figure 14. TEM images of HM-SiO_2 NPs before (**a**) and after (**b**) lyophilization. Scale bars, 100 nm. The size distribution (**c**) and ratio of full coating (**d**) of HM-SiO_2 NPs before and after lyophilization. Experiments in panels **a–c** were repeated three times independently with similar results. Data represent the mean ± s.d. (*n* = 3 independent experiments). Significance was determined by one-way ANOVA followed by post hoc Tukey test. ns: not significant. Source data are provided as a Source Data file.

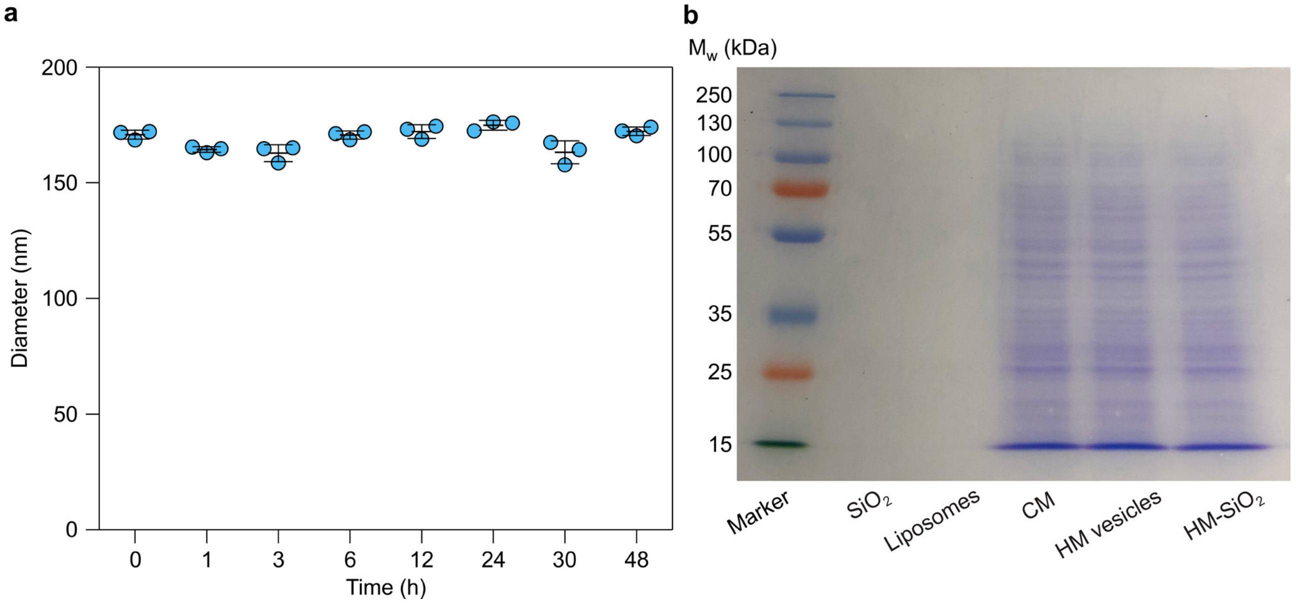

Supplementary Figure 15. a, Stability of HM-SiO_2 NPs in PBS at 37 °C was evaluated by monitoring the particle size changes at various time points up to 48 h. Data represent the mean ± s.d. (*n* = 3 independent experiments). **b,** SDS-PAGE protein analysis of SiO_2 NPs, liposomes, CM, HM vesicles, and HM-SiO_2 NPs. Experiments in panel **b** were repeated three times independently with similar results. Source data are provided as a Source Data file.

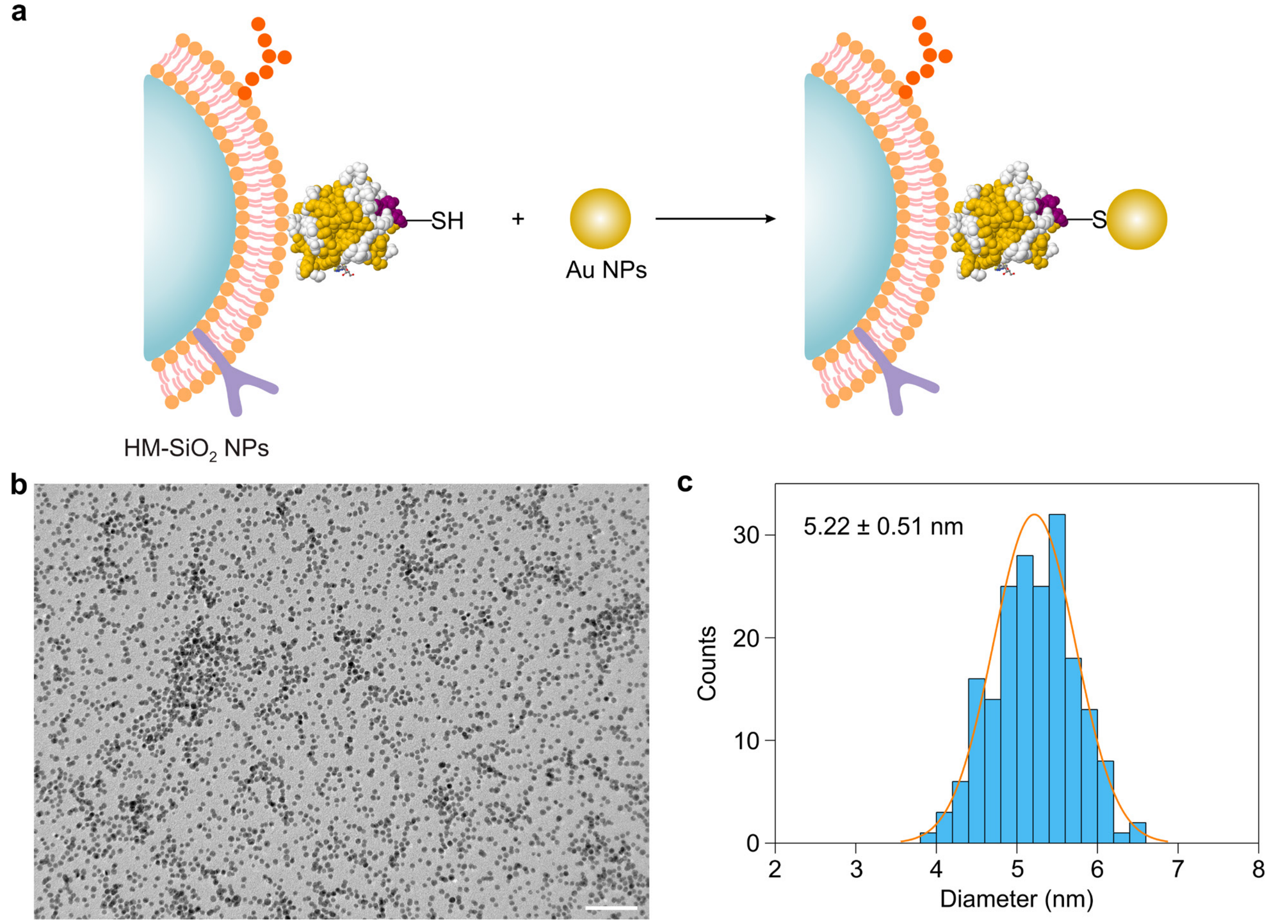

Supplementary Figure 16. a, Au NPs labeled-CM protein of HM-SiO_2 NPs were formed by the strong Au–S interactions between sulfhydryl group and Au NPs. TEM image (**b**) and size distribution (**c**) of Au NPs ($n = 192$ independent Au NPs). Scale bar, 50 nm. Experiments in panel **b** were repeated three times independently with similar results. Source data are provided as a Source Data file.

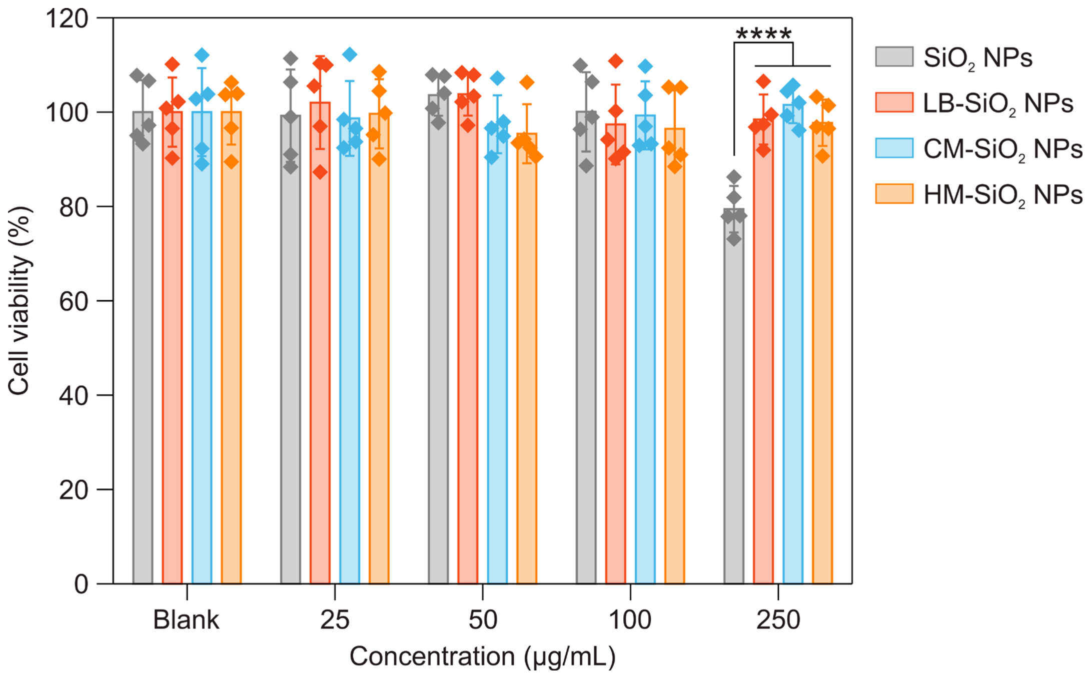

Supplementary Figure 17. Cell viability of CT26 cells after incubation with SiO_2 NPs, LB-SiO_2 NPs, CM-SiO_2 NPs, and HM-SiO_2 NPs for 24 h at different concentrations (25, 50, 100 and 250 μg/mL). Data represent the mean ± s.d. ($n = 5$ biologically independent cells). Significance was determined by one-way ANOVA followed by post hoc Tukey test. ****$p < 0.0001$. Source data are provided as a Source Data file.

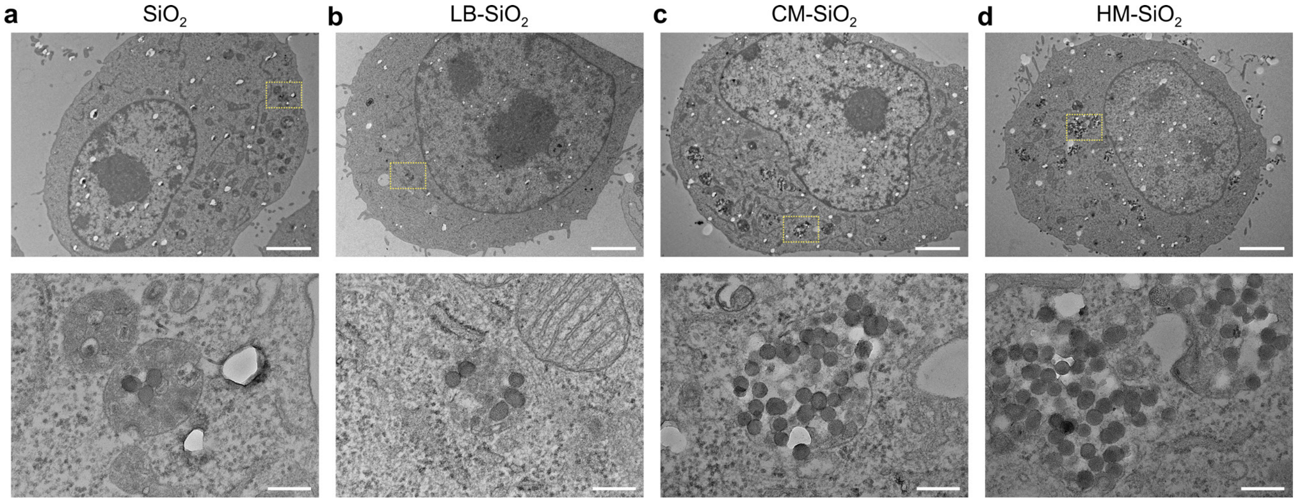

Supplementary Figure 18. TEM images of CT26 cells after 4 h incubation with SiO_2 NPs (**a**), LB-SiO_2 NPs (**b**), CM-SiO_2 NPs (**c**), and HM-SiO_2 NPs (**d**). Insets below are magnified of each image in the area highlighted with the respective yellow dashed box. Scale bars, 2 μm (top); 200 nm (bottom). Experiments were repeated three times independently with similar results.

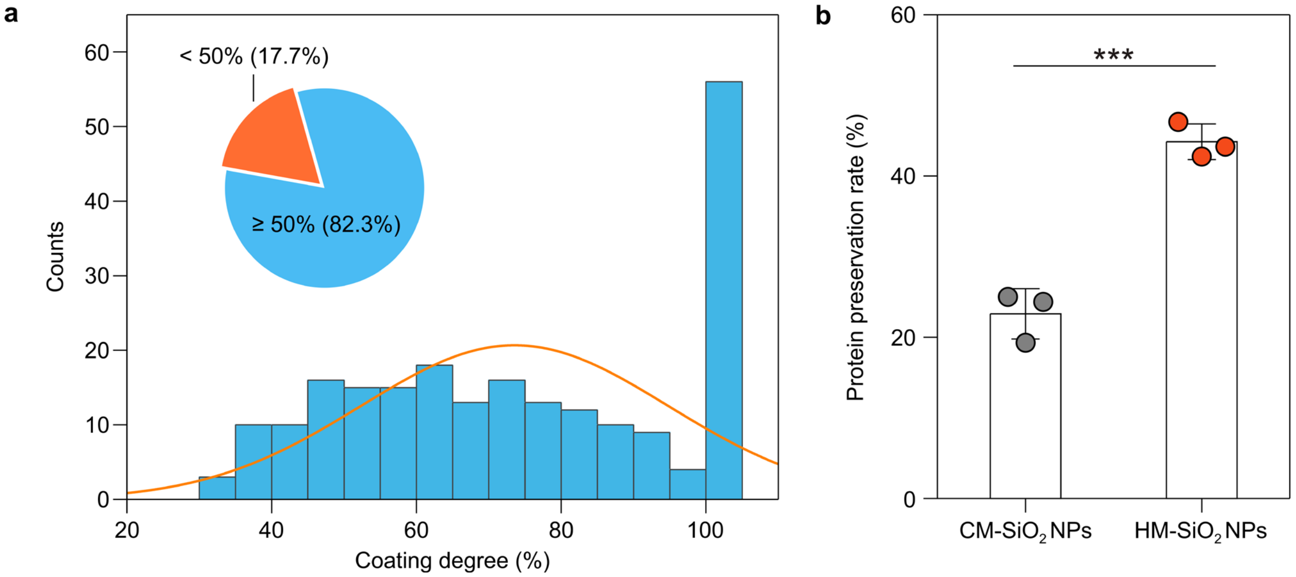

Supplementary Figure 19. a, CM coating degree distribution of HM-SiO_2 NPs, which is calculated from TEM images ($n = 220$ independent HM-SiO_2 NPs). The inset shows the proportion of SiO_2 NPs with a high CM coating degree (≥50%). **b,** Comparison of protein preservation rate of CM-SiO_2 NPs and HM-SiO_2 NPs when the same amount of protein was used to coat SiO_2 NPs. Data represent the mean ± s.d. (n = 3 independent experiments). Significance was determined by one-way ANOVA followed by post hoc Tukey test. $p = 0.0006$ (CM-SiO_2 NPs vs. HM-SiO_2 NPs). ***$p < 0.001$. Source data are provided as a Source Data file.

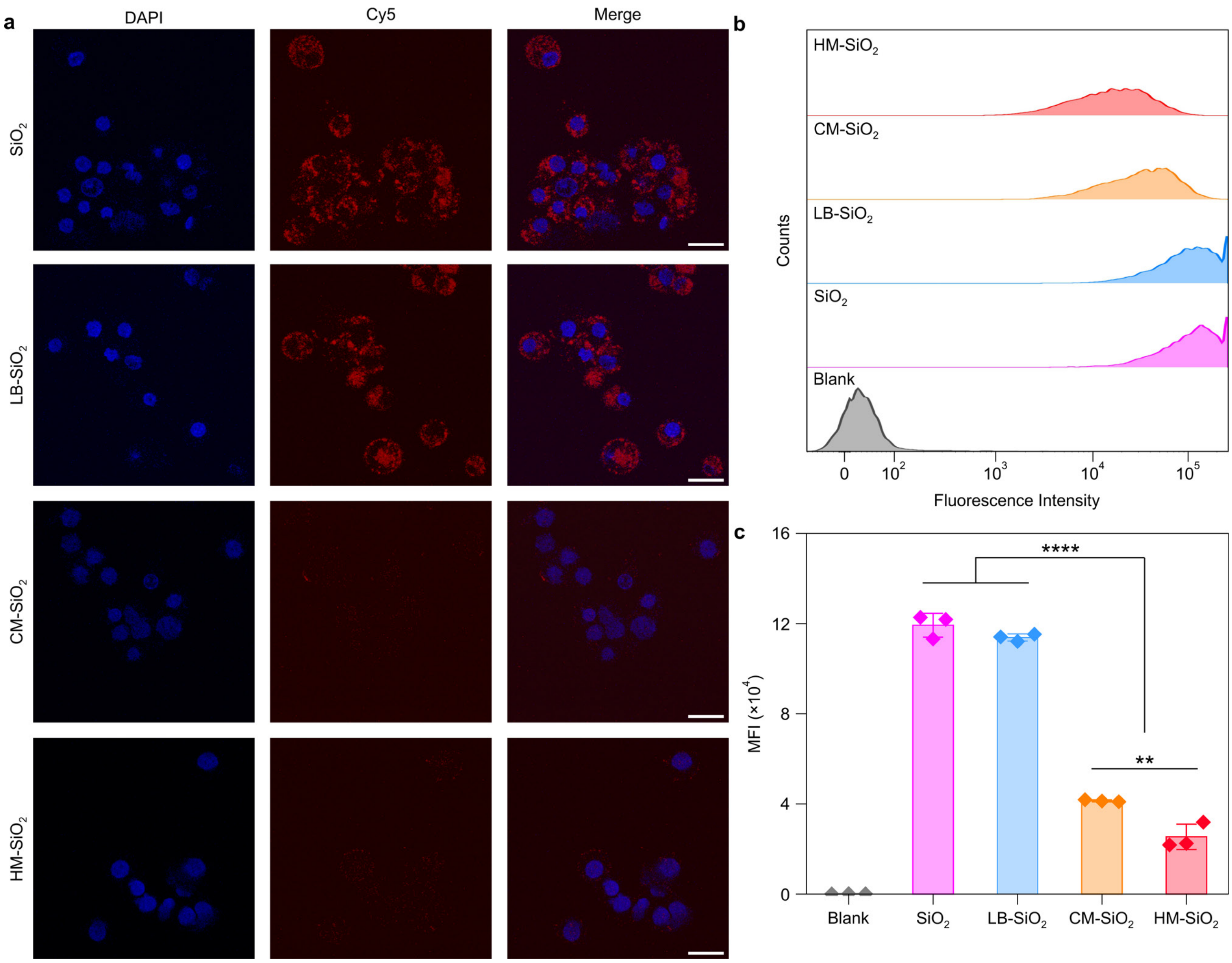

Supplementary Figure 20. a, Representative CLSM images of RAW264.7 cells after 4 h incubation with SiO_2 NPs, LB-SiO_2 NPs, CM-SiO_2 NPs, and HM-SiO_2 NPs. Blue, the cell nuclei stained with 4',6-diamidino-2-phenylindole (DAPI); red, Cy5 labeled SiO_2 cores. Scale bars, 20 μm. **b,** Flow cytometric analysis of RAW264.7 cells incubated with blank solution, SiO_2 NPs, LB-SiO_2 NPs, CM-SiO_2 NPs, and HM-SiO_2 NPs. **c,** Quantification of cellular uptake in RAW264.7 cells. Experiments in panels **a** and **b** were repeated three times independently with similar results. Data represent the mean ± s.d. (n = 3 biologically independent cells). Significance was determined by one-way ANOVA followed by post hoc Tukey test. $p = 0.00251$ (CM-SiO_2 NPs vs. HM-SiO_2 NPs). $^{**}p < 0.01$, $^{****}p < 0.0001$. Source data are provided as a Source Data file.

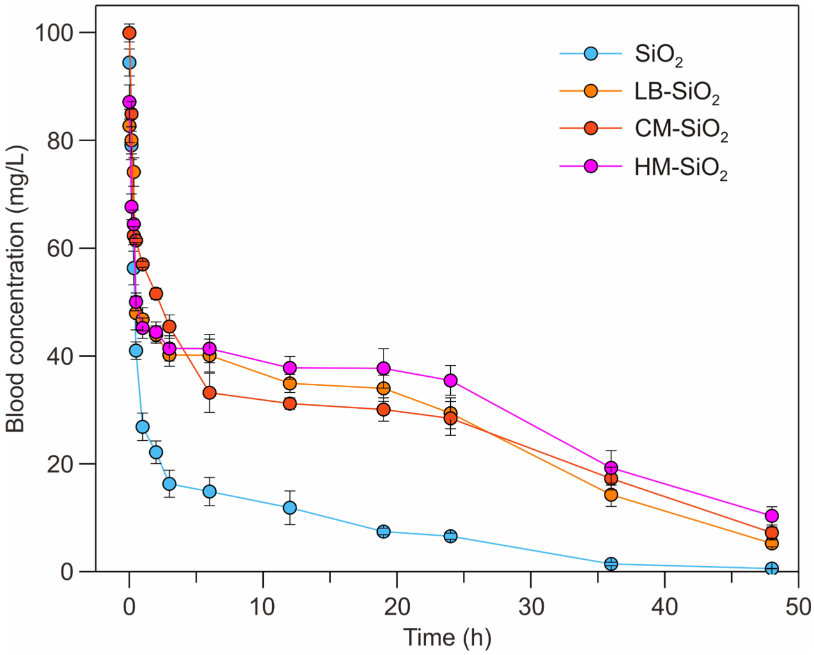

Supplementary Figure 21. Plasma concentration-time profiles of SiO_2 NPs in normal rats after intravenous injection of various formulations (Cy5 labeled-SiO_2 NPs, LB-SiO_2 NPs, CM-SiO_2 NPs, and HM-SiO_2 NPs) with the identical content of SiO_2 NPs at 6.25 mg/kg. Data represent the mean ± s.d. (n = 3 biologically independent rats). Source data are provided as a Source Data file.

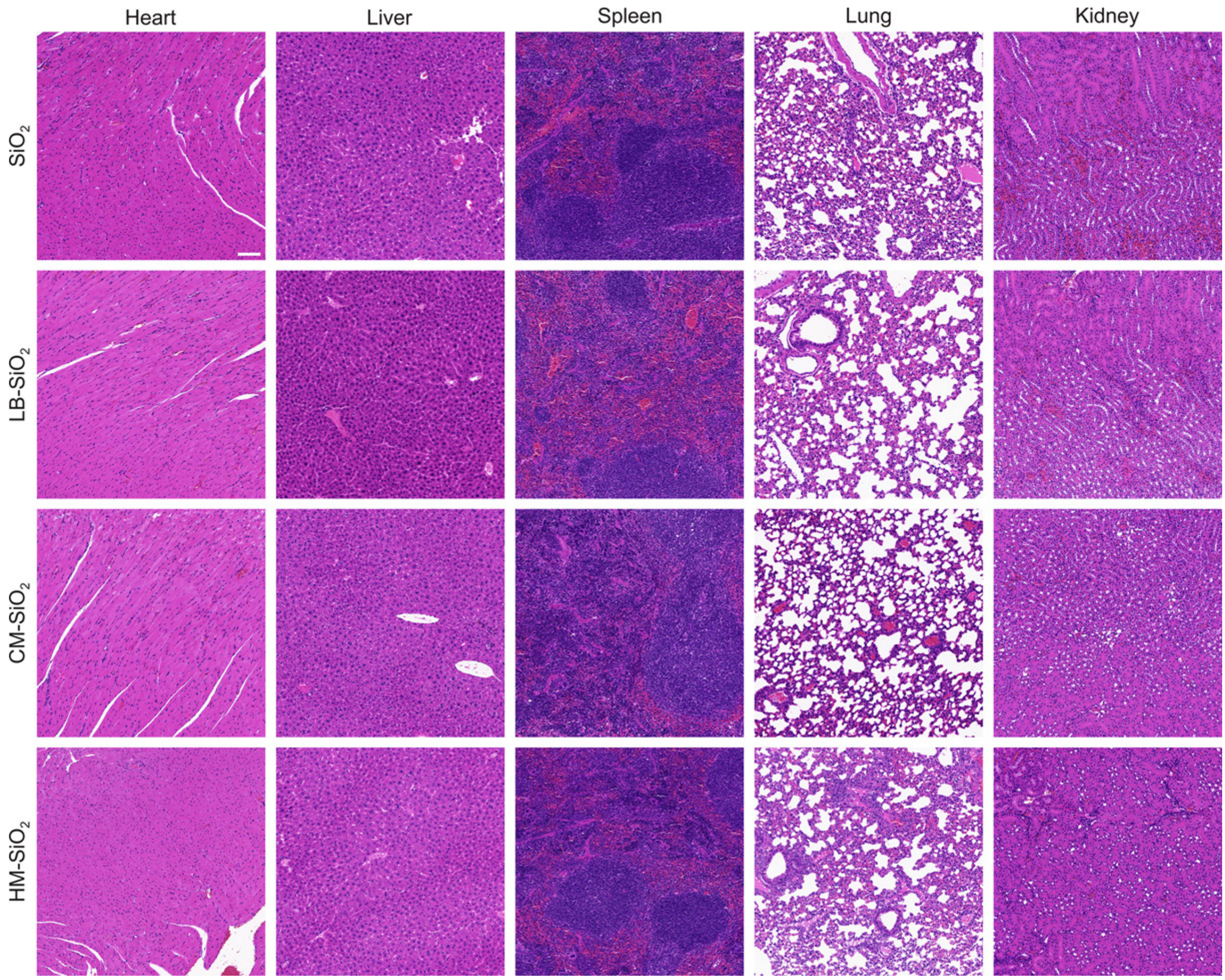

Supplementary Figure 22. H&E staining of heart, liver, spleen, lung, and kidney tissue slices for different treatment groups (SiO_2 NPs, LB-SiO_2 NPs, CM-SiO_2 NPs, and HM-SiO_2 NPs) on day 4. Scale bar, 100 μm. Experiments were repeated three times independently with similar results.

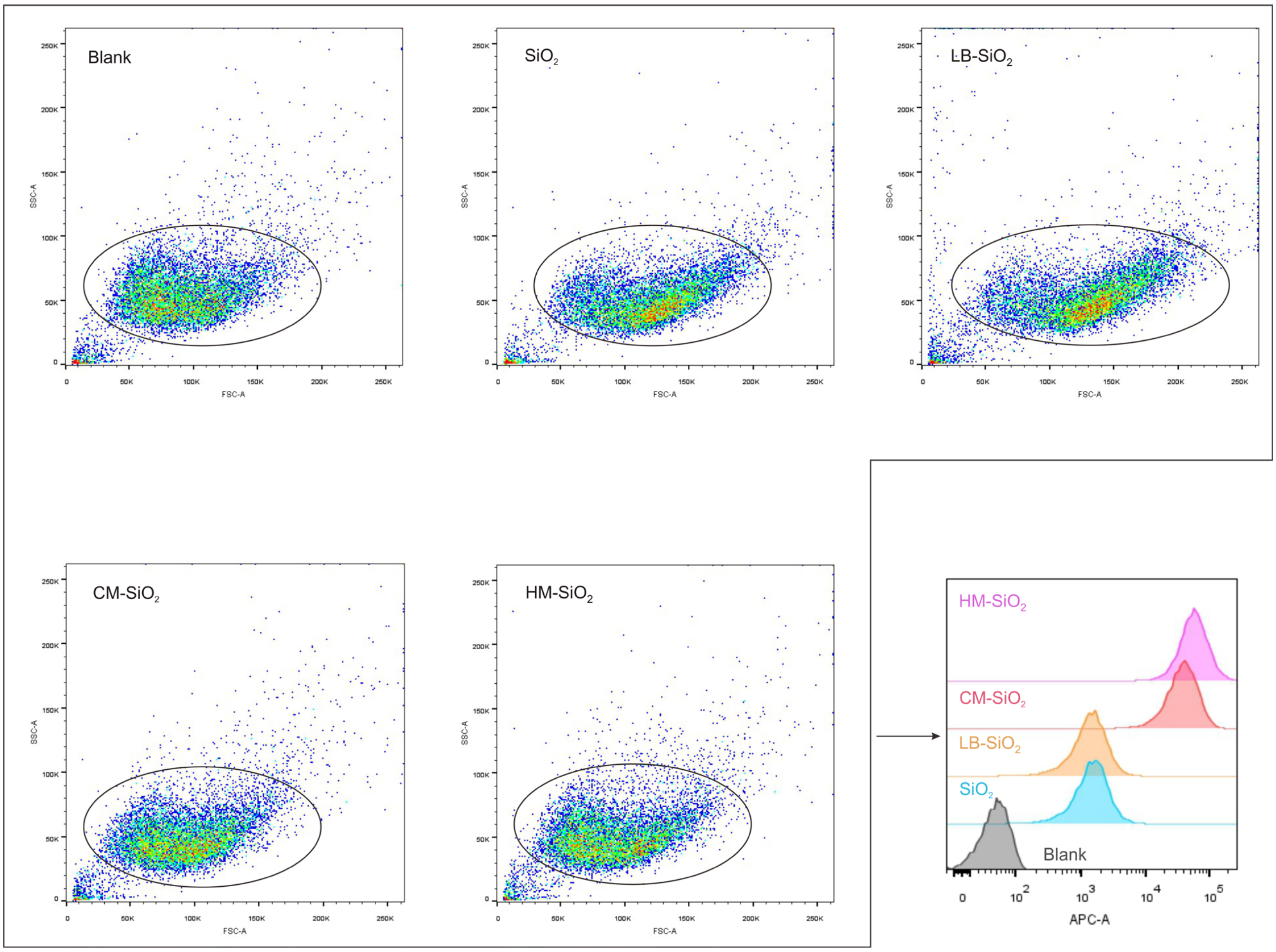

Supplementary Figure 23. Gating strategy to quantify NP binding and uptake *in vitro* (Fig. 5b-d, and Supplementary Fig. 20b). This experiment was repeated three times independently with similar results.

Supplementary Tables

Supplementary Table 1. Overview of the model parameters and values used in the Lattice Boltzmann method.

Parameter	Liposomes	CM vesicles	Source/method
Average size	100 nm	140 nm	DLS data
Average thickness (h)	4.16 nm	5.15 nm	Cryo-TEM images
Initial tension (σ_0)	10^{-7}~10^{-6} N/m	10^{-7}~10^{-6} N/m	Refs.[1, 2]
Elastic modulus (E)	0.9 MPa	3.6 MPa	AFM data
Expansion modulus (κ_a)	3.744 mN/m	18.54 mN/m	$k_a = E \times h$
Bending modulus (κ)	16.016 k_BT	121.61 k_BT	$\kappa = k_a \times h^2$
Pore size of the filter (D_h)	200 nm	200 nm	Product information
Inlet velocity (v)	10 cm/s	10 cm/s	Estimated from the extrusion process

Supplementary Table 2. Parameters for the theories of fixing pore.

Parameter	Value	Source/method
Line tension (Υ)	2.25×10^{-14} N	Estimated
Critical pore radius (r_c)	40 nm	Estimated
Initial surface tension (σ_i)	3.825×10^{-8} N/m	Estimated
Viscosity of internal liquid (η_0)	32 cP	Ref.[3]
Initial vesicle size (R_i)	90 nm	TEM image
Initial pore size	60 nm	TEM image
Thickness of the membrane (e)	10 nm	TEM image
Membrane viscosity (η_2)	1000 cP	Polarization measurements

Supplementary Table 3. Pharmacokinetic parameters of SiO_2 NPs after intravenous administration of Cy5 labeled-SiO_2 NPs, LB-SiO_2 NPs, CM-SiO_2 NPs, and HM-SiO_2 NPs with the identical content of SiO_2 NPs at 6.25 mg/kg (*n* = 3 biologically independent rats).

Formulations	Distribution half-life ($t_{1/2}α$, h)	Elimination half-life ($t_{1/2β}$, h)	Area under the curve ($AUC_{0-∞}$, mg/L×h)	Volume of distribution (V_d, L/kg)	Total body clearance (Cl, L/h/kg)	Mean residence time ($MRT_{0-∞}$, h)
SiO_2 NPs	0.25 ± 0.02	9.1 ± 0.8	360 ± 28	0.062 ± 0.006	0.017 ± 0.002	12.2 ± 1.1
LB-SiO_2 NPs	0.18 ± 0.01	16.2 ± 1.5	1178 ± 45	0.07± 0.005	0.005 ± 0.001	19.7 ± 1.9
CM-SiO_2 NPs	0.21 ± 0.02	18.5 ± 1.1	1345 ± 47	0.061 ± 0.006	0.005 ± 0.001	22.7 ± 3.3
HM-SiO_2 NPs	0.16 ± 0.02	23.6 ± 2.3	1578 ± 50	0.07 ± 0.007	0.004 ± 0.001	26.4 ± 2.5

Supplementary Notes

Supplementary Note 1. Calculation of the amount of lipid required for full coating of NPs

Theoretically, the amount of lipid required for single bilayer coverage of the NPs should be equal to the surface area of the core NPs[4]. Thus, the total surface area of 1 mg SiO_2 NPs can be calculated as follows:

Radius of the SiO_2 NP: $r = 35$ nm

Bulk density of the amorphous silica[5]: $\rho_b = 2.2$ g/cm^3

Porosity of mesoporous SiO_2 NPs: $\emptyset = 0.447$ (calculated from the Brunauer-Emmett-Teller data)

Envelope density of SiO_2 NPs: $\rho_e = \emptyset \times \rho_b$ =0.98 g/cm^3

Mass per SiO_2 NP: $M = \rho_e \times \frac{4}{3}\pi r^3 = 1.76 \times 10^{-16}$ g

Number of 1 mg SiO_2 NPs: $N = {}^{1\,mg}/_{M} = 5.68 \times 10^{12}$

Total surface area of 1 mg SiO_2 NPs: $S_{total} = N \times 4\pi r^2 = 8.74 \times 10^{16}\ nm^2$

Then, we estimated the surface area of DOPC liposomes by calculating the number of lipid molecules per unit mass and assumed 0.72 nm^2 to represent the headgroup area of DOPC[6]. In addition, the internal surface area is assumed to be equal to half the total surface area of the liposomes per unit mass[7]. The amount of lipid required for full coating of NPs was calculated as follows:

Headgroup area of DOPC: $A_{DOPC} = 0.72$ nm^2

Molecular weight of DOPC: $M_w = 786.113$ g/mol

Required DOPC amount: $m = \left(\frac{2 \times S_{total}}{A_{DOPC} \times N_A}\right) \times M_W = 0.32\ mg$

Supplementary Note 2. Investigation of pore fixing in CM vesicles

To gain deeper insights of the fixing process, we study the complete dynamic behavior of pores in vesicles, including pore opening driven by the membrane surface tension (σ), and the pore closure by the line tension (Υ). A rigorous analytical description of the process can be found in previous research[3]. Here, we briefly list the key expression from that work to explain our experiment observations. Assuming that there is only a single pore at a time in a given vesicle, and that during the opening and closure of a pore, the total amount of lipid is conserved, we can correlate the surface tension of the vesicle membrane (σ), the pore radius (r_p), and the radius of the vesicle (R_v) through the following expression[3]:

$$\frac{\sigma}{\sigma_i} = 1 - \frac{r_p^2}{r_c^2} - \frac{4\left(R_i^2 - R_v^2\right)}{r_c^2} \tag{1}$$

where r_c characterizes the critical size of the pore when the membrane tension of the vesicle is equal to zero, σ_i and R_i are the initial surface tension and radius of the vesicle without the pore. The line tension (Υ) of the pore edge drives the closure of the pore, the process of which can be formulated through[3, 8, 9]:

$$\frac{2\eta_2 e}{r_p}\frac{dr_p}{dt} = \sigma - \frac{\Upsilon}{r_p} \tag{2}$$

where η_2 defines the lipid viscosity, and e defines the thickness of the membrane. Equations above can be closed by considering the flux through the pore[3, 10]:

$$-4\pi R_v^2 \frac{dR_v}{dt} = \frac{2\sigma}{3\eta_0 R_v} r_p^3 \tag{3}$$

where η_0 is the viscosity of the solution inside the vesicle. To couple these three equations above, we can numerically calculate the fixing process of the pore for CM vesicles, which is shown in Fig. 3e. The related parameter settings are provided in Supplementary Table 2.

Supplementary References

1. Murrell, M. *et al.* Spreading dynamics of biomimetic actin cortices. *Biophys. J.* **100**, 1400-1409 (2011).
2. Fa, N., Marques, C.M., Mendes, E. & Schroder, A.P. Rheology of giant vesicles: a micropipette study. *Phys. Rev. Lett.* **92**, 108103 (2004).
3. Karatekin, E. *et al.* Cascades of transient pores in giant vesicles: line tension and transport. *Biophys. J.* **84**, 1734-1749 (2003).
4. Drazenovic, J., Ahmed, S., Tuzinkiewicz, N.M. & Wunder, S.L. Lipid exchange and transfer on nanoparticle supported lipid bilayers: effect of defects, ionic strength, and size. *Langmuir* **31**, 721-731 (2015).
5. Kruk, M., Jaroniec, M., Ko, C.H. & Ryoo, R. Characterization of the porous structure of SBA-15. *Chem. Mater.* **12**, 1961-1968 (2000).
6. Pichaandi, J. *et al.* Liposome-encapsulated $NaLnF_4$ nanoparticles for mass cytometry: evaluating nonspecific binding to cells. *Chem. Mater.* **29**, 4980-4990 (2017).
7. Durfee, P.N. *et al.* Mesoporous silica nanoparticle-supported lipid bilayers (protocells) for active targeting and delivery to individual leukemia cells. *ACS Nano* **10**, 8325-8345 (2016).
8. Sandre, O., Moreaux, L. & Brochard-Wyart, F. Dynamics of transient pores in stretched vesicles. *Proc. Natl. Acad. Sci. USA* **96**, 10591-10596 (1999).
9. Brochard-Wyart, F., de Gennes, P.-G. & Sandre, O. Transient pores in stretched vesicles: role of leak-out. *Phys. A* **278**, 32-51 (2000).
10. Happel, J. & Brenner, H. (Kluwer, Dordrecht, The Netherlands, 1983).
